# Radiotherapy for pelvic nodal recurrences after radical prostatectomy: patient selection in clinical practice

**DOI:** 10.1186/s13014-019-1383-0

**Published:** 2019-10-16

**Authors:** Cedric Panje, Thomas Zilli, Alan Dal Pra, Winfried Arnold, Kathrin Brouwer, Helena I. Garcia Schüler, Silvia Gomez, Fernanda Herrera, Kaouthar Khanfir, Alexandros Papachristofilou, Gianfranco Pesce, Christiane Reuter, Hansjörg Vees, Daniel Zwahlen, Paul Martin Putora

**Affiliations:** 1 Department of radiation oncology, Kantonsspital St. Gallen, Switzerland, Rorschacherstrasse 95, 9007 St. Gallen, Switzerland; 2 Department of radiation oncology, Hôpitaux Universitaires de Genève, Geneva, Switzerland; 3 Department of radiation oncology, Inselspital, Bern University Hospital, Bern, Switzerland; 4 Department of radiation oncology, Luzerner Kantonsspital, Lucerne, Switzerland; 5 Department of radiation oncology, Stadtspital Triemli, Zürich, Switzerland; 6 Department of radiation oncology, Universitätsspital Zürich, Zürich, Switzerland; 7 Department of radiation oncology, Kantonsspital Aarau, Aarau, Switzerland; 8 Department of radiation oncology, Centre Hospitalier Universitaire Vaudois, Lausanne, Switzerland; 9 Department of radiation oncology, Hôpital du Valais, Sion, Switzerland; 10 Department of radiation oncology, Universitätsspital Basel, Basel, Switzerland; 11 Department of radiation oncology, EOC Bellinzona, Bellinzona, Switzerland; 12 Department of radiation oncology, Kantonsspital Münsterlingen, Münsterlingen, Switzerland; 13 Department of radiation oncology, Klinik Hirslanden, Zürich, Switzerland; 14 Department of radiation oncology, Kantonsspital Graubünden, Chur, Switzerland; 15 Department of radiation oncology, University of Bern, Bern, Switzerland

**Keywords:** Nodal recurrence, Prostate cancer, Oligometastatic, Radiotherapy, SBRT, Decision making, Decision criteria

## Abstract

**Aim:**

There is no general consensus on the optimal treatment for prostate cancer (PC) patients with intrapelvic nodal oligorecurrences after radical prostatectomy. Besides androgen deprivation therapy (ADT) as standard of care, both elective nodal radiotherapy (ENRT) and stereotactic body radiotherapy (SBRT) as well as salvage lymph node dissection (sLND) are common treatment options. The aim of our study was to assess decision making and practice patterns for salvage radiotherapy (RT) in this setting.

**Methods:**

Treatment recommendations from 14 Swiss radiation oncology centers were collected and converted into decision trees. An iterative process using the objective consensus methodology was applied to assess differences and consensus.

**Results:**

PSMA PET/CT was recommended by 93% of the centers as restaging modality. For unfit patients defined by age, comorbidities or low performance status, androgen deprivation therapy (ADT) alone was recommended by more than 70%. For fit patients with unfavorable tumor characteristics such as short prostate-specific antigen (PSA) doubling time or initial high-risk disease, the majority of the centers (57–71%) recommended ENRT + ADT for 1–4 lesions. For fit patients with favorable tumor characteristics, there were low levels of consensus and a wide variety of recommendations. For 1–4 nodal lesions, focal SBRT was offered by 64% of the centers, most commonly as a 5-fraction course.

**Conclusions:**

As an alternative to ADT, ENRT or SBRT for pelvic nodal oligorecurrences of PC are commonly offered to selected patients, with large treatment variations between centers. The exact number of lymph nodes had a major impact on treatment selection.

## Background

Regional nodal recurrence of prostate cancer (PC) limited to the pelvis (pelvic oligorecurrences) after curative local therapies such as radical prostatectomy (RP) or primary radiotherapy (RT) is an emerging clinical scenario. In part, this may be explained by the broad implementation of novel metabolic imaging strategies such as choline and prostate specific membrane antigen (PSMA) PET-CT in the last years [1–3]. These new imaging modalities have improved both sensitivity and specificity to detect the site of tumor recurrence in case of rising prostate-specific antigen (PSA) leading to an increased utilization of metastasis-directed approaches. Nevertheless, metastasis-directed therapies (MDT) such as stereotactic body radiotherapy (SBRT), salvage lymph node dissection (sLND) or elective nodal radiotherapy (ENRT) to the pelvis remain a controversial issue as an addition or replacement therapy modality to androgen deprivation therapy (ADT) [2].

Current PC guidelines such as the European Association of Urology (EAU) and European Society of Radiation Oncology (ESTRO) Guidelines [4] do not specifically address pelvic oligorecurrences, as there is very limited data from prospective trials [2, 5, 6]. In contrast, there is an increasing number of retrospective studies suggesting that patients with pelvic oligorecurrences may benefit from MDT [1, 7, 8]. Optimal patient selection for loco-regional therapy based on the number of nodal recurrences and other risk factors is currently unclear.

Based on similar analyses among radiation oncology centers on radiotherapy for primary PC and for macroscopic local recurrences [9, 10], the aim of this study was to assess current patterns of practice for pelvic nodal oligorecurrences of PC after RP among centers within a similar environment. The Swiss centers that were analyzed are all within the same environment characterized by universal healthcare coverage, modern equipment, uncomplicated reimbursement and no significant logistical or transport obstacles for patients.

## Methods

We contacted all Swiss university hospitals (*n* = 5) and radiation oncology centers (*n* = 9) which had participated in the Swiss Group for Clinical Cancer Research (SAKK) prospective study 09/10 on dose-escalated salvage radiotherapy for biochemically recurrent disease [11] in congruence with a previous survey [9].

Representatives from fourteen centers were asked to provide their institutional treatment recommendations in any format (e.g. figure, text, diagram), which were consequently converted into decision trees in a bilateral iterative process between the coordinating center and the participants (objective consensus methodology) as previously described [9, 12, 13]. Specific decision criteria or cut-off values were not provided to avoid bias. Additionally, the representatives were asked to define target volume definitions and planning target volume (PTV) margins, radiation dose prescription and describe their use of concomitant ADT. Institutional treatment recommendations were collected as free unrestricted text until June 2019. Consequently, they were converted into decision trees (by CP and PMP) and verified in a bilateral process by the individual participants. In order to improve comparability of treatment recommendations, standardized common decision criteria for tumor characteristics and patient fitness were established and accepted by all participating centers [12]. The initial open-question survey which was sent to the participating centers can be found in the Additional file 1.

**Fig. 1:**
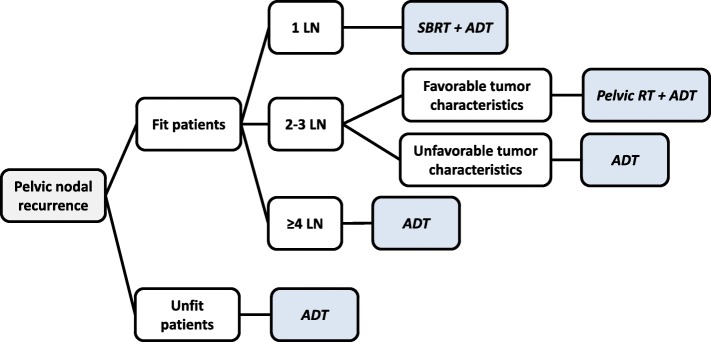
Representative decision tree from a single participating center. LN, lymph node; RT, radiotherapy; SBRT, stereotactic body radiotherapy; ADT, androgen deprivation therapy

The resulting treatment algorithms were compared semi-automatically and analyzed for consensus and differences [13]. A representative treatment decision tree of a participating center is shown in Fig. 1.

## Results

Fourteen Swiss radiation oncology centers were contacted. All centers returned the survey and the review of the center-specific treatment algorithms were confirmed in June 2019.

Decision criteria from all centers were collected and merged with the agreement of all participants into the criteria “patient fitness” and “favorable vs. unfavorable tumor characteristics” in order to facilitate the comparability of decision algorithms [12]: Patient fitness was defined by the majority of centers by age (64%) and performance status (57%) as well as by comorbidities (64%). However, specific cut-off values or exclusion criteria for these factors were not provided and the final decision on patient fitness was left to the discretion of the treating physician in all centers. Tumor characteristics considered unfavorable and relevant for treatment decisions were inconsistently defined among the centers and are summarized in Table 1.

**Table 1:** Tumor characteristics named as relevant for treatment decision

Characteristic	% (number of centers)
PSA doubling time	57% (*n* = 8)
Initial high-risk disease	36% (n = 5)
PSA level at recurrence	29% (n = 4)
Size of lymph node recurrence	14% (n = 2)
Interval since RP	14% (n = 2)

Multiple factors were named by some centers. PSA, prostate-specific antigen. RP, radical prostatectomy.

**Fig. 2:**
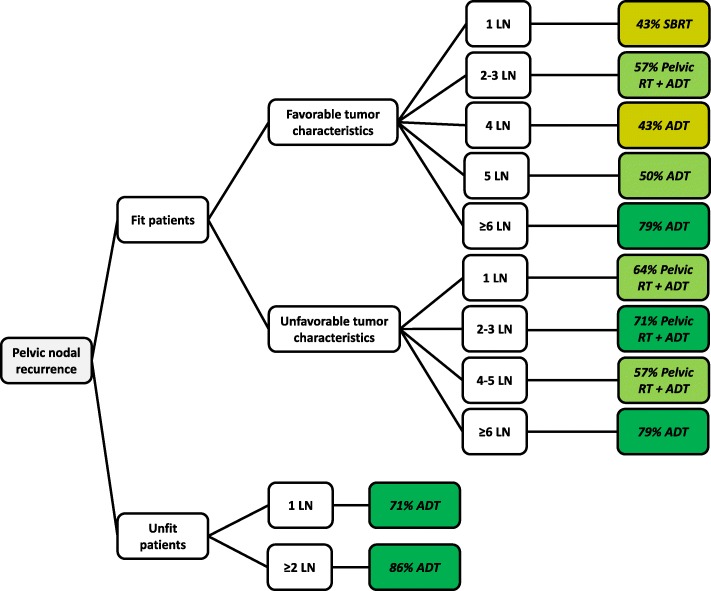
Mode decision tree for intra-pelvic lymph node recurrences of prostate cancer after RPE. LN, lymph node; RT, radiotherapy; SBRT, stereotactic body radiotherapy; ADT, androgen deprivation therapy

The highest level of consensus for the use of any RT (71%) was obtained for the use of pelvic RT and ADT in fit patients with unfavorable tumor characteristics and two to three lymph node recurrences (Fig. 2). There was a high level of consensus for the use of ADT without RT in case of six or more nodal recurrences in fit patient or in two or more nodal recurrences in unfit patients.

Fit patients with favorable tumor characteristics having 1 or 4 lymph node recurrences lead to the most heterogeneous recommendations. The variety of treatment recommendations for these two scenarios are shown in Table 2.

**Table 2:** Treatment recommendations for clinical scenarios without consensus

Scenario without consensus	Recommended treatment options (number of centers)
Single lymph node recurrence Fit patient Favorable tumor characteristics	43% SBRT (n = 6) 29% Pelvic RT + ADT (*n* = 4) 7% SBRT + ADT (*n* = 1) 7% SBRT or pelvic RT (*n* = 1) 7% SBRT +/−ADT or pelvic RT + ADT (n = 1) 7% Surgery and/or pelvic RT + ADT (n = 1)
Four lymph node recurrences Fit patient Favorable tumor characteristics	43% ADT (*n* = 6) 36% Pelvic RT + ADT (*n* = 5) 14% SBRT (*n* = 2) 7% Pelvic RT (n = 1)

RT, radiotherapy; SBRT, stereotactic body radiotherapy; ADT, androgen deprivation therapy.

Treatment specifications are summarized in Table 3. PSMA PET/CT was recommended by all but one center as the standard restaging imaging modality (93% of the centers). An additional multiparametric pelvic magnetic resonance imaging (MRI) was recommended by 64% of the centers.

**Table 3:** Treatment specifications

Factor	Recommendations
Recommended Imaging before therapeutic decision	93% PSMA PET/CT
	64% pelvic multiparametric MRI
	further: bone scan, PSMA-PET MRI, choline PET (1 center each)
Dose to elective pelvic lymph nodes (ENRT)	Median dose 50 Gy (range, 45–54 Gy) in 1.8–2 Gy/fraction
	No pelvic RT recommended by 14%
Prostate bed RT	Always included by 58% of the centers using pelvic RT
	Median dose 66 Gy (range, 64–70 Gy)
Dose for lymph node boost for pelvic RT	SIB in 75%, median dose 66 Gy (range 57.5–70 Gy, single dose 2–2.5 Gy)
	SBRT boost in 25% (2 × 5 Gy)
SBRT*	29% 3-fraction course (SD 10–15 Gy)
	43% 5-fraction course (SD 6–8 Gy)
	14% > 10 fraction course (SD 3.5 Gy)
	36% no primary SBRT
Margins SBRT	CTV, median 0 mm (range, 0–5 mm)
	PTV, median 5 mm (range, 3–5 mm)
Recommendation for concomitant ADT	standard duration 57% six months, 7% nine months
	21% in the presence of risk factors 6–24 months
	29% no concomitant ADT (in addition to SBRT)

* for SBRT, some center provided more than one fractionation schedule

CTV, clinical target volume; PSMA, prostate specific membrane antigen; PTV, planning target volume; RT, radiotherapy; SIB, simultaneous integrated boost; SBRT, stereotactic body radiotherapy; SD, single dose.

Median dose recommendation for ENRT was 50 Gy, with inclusion of the prostate bed in 58% of the centers (*n* = 7). SBRT for pelvic lymph node recurrences was most commonly recommended in a five-fraction course with daily doses of 6 to 8 Gy.

The majority of centers (57%) recommended the addition of concomitant ADT for a duration of six months and one center for nine months (7%). Twenty-one percent of the centers (*n* = 4) recommended ADT in the presence of risk factors for up to 24 months. One center left the duration of the ADT to the discretion of the referring urologist.

## Discussion

A proportion of patients with prostate cancer develop local, regional or distant recurrence after curative local treatment, which is increasingly detected due to novel imaging modalities such as choline or PSMA PET-CT [1, 3]. PC patients with pelvic oligorecurrences show a more favorable prognosis than patients with recurrent visceral or bone metastases [1]. However, this circumstance is not addressed in current guidelines for stage IV prostate cancer [4]. Although high-level evidence for local therapies in oligorecurrent prostate cancer is still lacking, both MDT such as SBRT or sLND and ENRT are increasingly in use [2], mainly based on suggestive data from retrospective series [14]. Additionally, there is increasing evidence on the benefits of MDT in oligometastatic disease in other tumor entities: The recently published randomized phase II SABR-COMET trial demonstrated an increased OS (41 vs. 28 months) for SBRT in addition to standard systemic therapy in oligometastatic patients with different primaries, also including prostate cancer [15]. In stage IV non-small cell lung cancer, two randomized phase II trials reported for local therapy to oligometastatic sites, including SBRT, an improved PFS as well as improved OS in one of the trials [16, 17].

Our analysis shows that loco-regional treatment is commonly offered to patients with intrapelvic oligorecurrent prostate cancer, but there is considerable variety in the choice of the specific treatment modality. We would expect that the rather homogeneous environment of the healthcare system in Switzerland would not cause differences in treatment recommendations due to the lack of resources, insurance coverage or available technology [18].

With respect to imaging modalities, all centers recommended choline or PSMA PET-CT which is currently common practice in case of biochemical recurrence after primary curative therapy of prostate cancer [2, 19]. A recent study on the impact of PSMA PET-CT on treatment decisions for recurrent prostate cancer showed that patient management changed in 60% with a substantial increase in metastasis-directed treatment and a reduction in the use of systemic therapy [20]. These findings are in line with a prospective study investigating the impact of PSMA PET-CT on the management of patients with a biochemical recurrence of prostate cancer. PSMA PET-CT changed management in 54 of 101 patients (53%). PSMA PET-CTs in a series of 125 patients detected recurrences, which would be missed by standard radiation fields to the prostate bed in every third patient [21].

In “unfit” patients defined by age, performance status or comorbidities, more than two thirds of the centers recommended ADT alone without further local therapy irrespective of the number of lymph node recurrences. Also, 79% of the centers recommended ADT alone for more than five lymph node recurrences irrespective of other tumor characteristics. However, we did not include the recommended time point of the start of ADT (immediate versus delayed) in our survey, as this treatment decision is most commonly taken by the responsible urologist or medical oncologist in Switzerland. For patients with biochemical recurrence of prostate cancer, delaying ADT is a valuable option. The authors believe this is especially the case when rapid disease progression is not expected and in cases of severe comorbidities potentially complicating earlier treatment, as ADT may worsen the quality of life and may aggravate cardiovascular morbidity [4]. This being said, optimal patient selection remains a challenge.

Three to five lesions are often considered the upper limit of oligometastatic disease that may benefit from additional local treatment [2]. In the postoperative setting of node positive (pN1) prostate cancer, Abdollah et al. showed in a large retrospective analysis that adjuvant radiotherapy was associated with better survival only in patients with up to four lymph nodes [22].

In summary, ADT with or without MDT was recommended by the majority of centers for pelvic oligorecurrences according to current guidelines for stage IV disease [4]. However, for fit patients with favorable tumor characteristics and a single lymph node recurrence, 50% of the centers recommended MDT (mainly SBRT) without concomitant ADT. For unfit patients, SBRT alone for single lymph node recurrences was an option for 29% of the centers. This strategy may be supported by the recent randomized phase II STOMP trial, which showed that MDT in oligorecurrent prostate cancer increased the median ADT-free survival from 13 months to 21 months [5], although one should note that in our analysis ADT was typically recommended concurrently. Likewise, the Australian phase I POPSTAR trial showed that single fraction SBRT is safe and leads to a 2-year freedom of ADT in 48% of the patients [6]. While this finding seems reproducible, in many cases the right timing to start ADT remains unclear.

Interestingly, one radiation oncology center recommended sLND as a treatment option for a single lymph node recurrence in fit patients with favorable tumor characteristics as alternative to SBRT [23].

In contrast, for patients with unfavorable tumor characteristics, more than half of the centers recommended ENRT plus ADT.

Currently it remains unclear whether MDT such as sLND or SBRT provide sufficient control for intrapelvic recurrences of prostate cancer. Although direct comparative data is scarce, there is a trend for longer progression-free survival (PFS) rates in patients receiving ENRT of the pelvis [24] compared to focal SBRT [25, 26] or sLND alone [23]. Rischke et al. [27] reported on a series of 93 patients with pelvic lymph node recurrences which received sLND with or without ENRT. Five-year biochemical PFS rates were significantly higher in patients receiving additional RT (34.3% vs. 15.4%) [27]. Similarly, Lepinoy et al. compared 35 patients receiving SBRT to 27 patients receiving ENRT for nodal oligorecurrences from prostate cancer [28]. Three-year failure rate was significantly higher after MDT (88.3% vs. 55.3%) without increased late toxicity in the ENRT group [28]. In summary, the available retrospective series on ENRT versus SBRT report improved PFS for ENRT, although there is the potential risk of increased toxicity by ENRT compared to SBRT [29].

Based on this preliminary evidence, it appears reasonable that most centers recommended ENRT in patients with unfavorable tumor characteristics with a higher risk of disease progression. MDT (SBRT or sLND) and ENRT (exclusive or adjuvant after sLND) for three or less oligorecurrent pelvic lymph node recurrences of prostate cancer in addition to short-term ADT are currently prospectively compared in the ongoing randomized phase II STORM trial (NCT03569241). Additionally, the GETUG OLIGOPELVIS P07 trial (NCT02274779) will prospectively evaluate the use of high-dose ENRT plus ADT in oligorecurrent prostate cancer with up to five lymph nodes [30].

Patient selection for MDT or ENRT remains a critical issue. In our survey, most experts used PSA-related factors such as PSA level, PSA doubling time and time from RP to biochemical progression as clinical surrogate parameters for adverse tumor biology as commonly done in other disease stages in prostate cancer [31, 32]. Also, the initial risk group defined by the T category of the primary tumor, the histological Gleason score and the initial PSA [31] was explicitly considered by more than one third of the centers. For patients with these unfavorable tumor characteristics, the majority of the centers recommended more commonly ENRT combined with ADT instead of MDT alone. Patients with these adverse features are considered at a higher risk for rapid loco-regional or systemic progression. Tran et al. showed that oligorecurrent patients with five or less nodal metastases had a significantly reduced 5-year PFS (36.8% vs. 63.6%) in case of a PSA doubling time of less than 3 months even when ENRT plus ADT was used [24].

Additional translational research is needed in order to improve our understanding of tumor biology of oligorecurrent prostate cancer and to optimize risk stratification and patient selection for each of the available salvage therapies [2].

Our study had some inherent limitations. While we assume that we captured a representative picture of the current patterns of practice, not every patient may be treated according to the collected algorithms, as treatment decisions in oligometastatic disease are commonly a result of an interdisciplinary discussion of the radiation oncologist with the referring urologist or medical oncologist under consideration of the patients’ individual preferences and risks. Additionally, we cannot exclude that other factors may influence decisions, which have not been considered in our analysis [33]. Also, our survey may have a “specialty bias”, as radiation oncologists like other specialists tend to recommend their own treatment options more frequently [34, 35].

## Conclusions

In conclusion, the use of MDT strategies like SBRT and ENRT has a high acceptance among radiation oncologists and are commonly recommended for PC patients with pelvic oligorecurrences outside of clinical studies. However, treatment recommendations are very heterogeneous among centers, without a clear consensus. The exact number of lymph nodes was a very influential decision criterion in treatment selection. Ongoing prospective trials will hopefully provide further answers to open questions, and improve the evidence on the best treatment modality for oligorecurrent PC patients.

## Additional file

**Additional file 1.** Initial open-question survey which was sent to the participating centers.

## Data Availability

The datasets used and analyzed during the current study are available from the corresponding author on reasonable request.

## Abbreviations

ADT: Androgen deprivation therapy CT Computed tomography
CTV: Clinical target volume
ENRT: Elective nodal radiotherapy
MRI: Magnetic resonance tomography
MTD: Metastasis-directed therapy
OS: Overall survival
PC: Prostate cancer
PET: Positron emission tomography
PFS: Progression-free survival
PSA: Prostate specific antigen
PSMA: Prostate specific membrane antigen
PTV: Planning target volume
RP: Radical prostatectomy
RT: Radiotherapy
SBRT: Stereotactic body radiotherapy
SD: Single dose
SIB: Simultaneous integrated boost
sLND: Salvage lymph node dissection
