# Supplementary material for: Radiotherapy for pelvic nodal recurrences after radical prostatectomy: patient selection in clinical practice
Source: Radiat Oncol. 2019 Oct 16;14:177. doi: 10.1186/s13014-019-1383-0 (PMC6796467; doi:10.1186/s13014-019-1383-0)
Supplement: Supplementary file 1 — Additional file 1. Initial open-question survey which was sent to the participating centers. [file 13014_2019_1383_MOESM1_ESM.docx]

**Initial open-question survey which was sent to the participating centers**

*“Please describe your management of patients with oligorecurrent prostate cancer after primary radical prostatectomy for intrapelvic nodal recurrence (no distant metastases, no local recurrence, ADT-naïve).*

*Please describe all criteria which are relevant to your decision.*

*Also, please describe if you have any requirements for imaging/verification. Please also describe the specifics of your target volumes/dose.”*
